# How Do Mental Health Nursing Students in the United Kingdom Experience Assessment Against the NMC Generic Standards of Proficiency? A Cross‐Field Comparison

**DOI:** 10.1111/inm.70133

**Published:** 2025-09-05

**Authors:** Mark Kenwright, Carolyn McCrorie, Christopher Bye, Patricia Awty, Donna Doherty, Maxine Cromar‐Hayes

**Affiliations:** ^1^ Centre for Health Innovation University of Staffordshire Stafford UK; ^2^ University of Huddersfield Huddersfield UK

**Keywords:** curriculum, field‐specific, generic, mental health nursing, proficiencies

## Abstract

In 2018 the UK's Nursing and Midwifery Council (NMC) moved from field‐specific to generic standards of proficiency in training all four fields of nursing practice. Some educators claim these proficiencies exclude field‐specific content, but there is a lack of comparative evidence on student feedback across each field. This study examined student nurses experiences of being assessed against these generic proficiencies to identify whether there are any differences between the fields of nursing in the United Kingdom. In a cross‐sectional study employing a mixed‐methods anonymous online survey, 531 nursing students in the final 3 months of training were surveyed across UK universities in 10 regions of the United Kingdom. Significant differences between nursing fields were observed in students' feedback on the NMC proficiencies (*p* = 0.000). Mental health nursing students reported the lowest levels of practice confidence; significant barriers to achieving proficiencies; and exclusion of field‐specific knowledge and skills, which devalued and threatened their professional identity. Adult nursing students reported high levels of practice confidence; fewer barriers to achieving proficiencies; variable experiences of placement support; and a preference for more focus on communication skills. Feedback indicates that students experience the generic proficiencies as a list of medical procedures that exclude communication and relationship management skills. The findings suggest that further evaluation and guidance are required to ensure that mental health proficiencies are included in practice assessment documentation.

## 1 Introduction

The United Kingdom currently retains four distinct fields of nursing practice: Adult; Mental Health; Children's and Learning Disabilities—each with its own route to registration with the Nursing and Midwifery Council (NMC). Up until 2018, students in each of these fields were taught and assessed against separate proficiencies (NMC 2010). In 2018, the NMC introduced generic standards of proficiency across all fields of nursing (NMC 2018a), which were accompanied by new standards for education and training, giving UK universities the flexibility and autonomy to decide on the proportion of generic and field‐specific content/hours provided (NMC 2018b).

The standards state that the level of knowledge and expertise required for each procedure will vary depending on the field of nursing being studied, meaning that students should demonstrate, ‘the ability to undertake these procedures at an appropriate level for their intended field(s) of practice’ (NMC 2018a). However, critics suggest that universities are not demonstrating this field‐specific flexibility, with claims that nurse training has become too generic and overly focused on adult medical procedures (Glasper and Fallon 2021; Haslam 2023; Bifarin et al. 2024; Evans 2024; Whaley et al. 2024).

## 2 Background

Concerns about a reduction in field‐specific content have been raised by educators in all fields outside of adult nursing. Children's nursing academics describe a reduction in child specific content within nursing curriculums across UK universities (Glasper and Fallon 2021; Fallon 2023; Reynolds et al. 2024) as a serious concern, arguing that addressing the biopsychosocial needs of children, young people and their families requires specialist knowledge and skills (Tatterton et al. 2024), such as child development (Glasper and Charles‐Edwards 2002). The learning disabilities nursing field has raised similar concerns about generic nurse education being insufficient to meet the complex health and social care needs of people with learning disabilities (Cogher 2023). The field of mental health nursing has been the most actively critical of the generic standards, describing them as an ‘Assault’ on the field (Warrender 2022). This has led to the formation of a grass roots campaign group ‘Mental Health Deserves Better’ who wrote an open letter to the NMC expressing concerns that the standards of proficiency prescribe a generic nurse education syllabus (Mental Health Deserves Better 2023). Haslam (2023) has argued that this has, ‘devalued the unique position and skill set of the mental health nurse’ and other mental health nursing academics have expressed concerns that the proficiencies dilute the mental health nursing role in favour of a generic nurse, which threatens the identity and existence of mental health nursing as a professional field (Connell et al. 2022; Haslam 2023, 2025; McKeown 2023; Warrender et al. 2023). Despite Health Education England publishing a framework of field specific competencies for mental health nursing as a distinct profession (Health Education England 2020, 2022), it is argued that these are missing from the NMC generic proficiencies (Haslam 2023; Warrender et al. 2023).

The move towards genericism by the UK's NMC appears to be following the direction taken by other countries such as Australia, where the move to a generic nursing curriculum and qualification has led to a de‐emphasis on mental health skills resulting in workforce shortages, reduced quality of care and ultimately worse outcomes for service users (Lakeman et al. 2024; Hurley and Lakeman 2021; Lakeman and Molloy 2018; Sheehan et al. 2025). Initial evidence on the effect of generic standards in the United Kingdom has suggested a lack of consistency and agreement over what constitutes a sufficient level of proficiency across different fields of practice (Bifarin et al. 2024) with claims of nursing students chasing specific medical proficiencies that some practice assessors are unwilling to sign off because they are rarely practised in their field (Painter and Bond 2023). Seven years on from their launch, the only published evaluation of the generic standards of proficiency in the United Kingdom collates the views of 199 educators and students without mention of the specific fields of nursing (Whaley et al. 2024). The NMC and UK Chief Nursing officers response to the open letter from ‘Mental Health Deserves Better’ included the statements, ‘Approved Education Institutions may seek views on the delivery and effectiveness of their co‐produced curricula in meeting field specific requirements from stakeholders, including students… which the NMC would welcome hearing… we are collectively committed to hearing the views of stakeholders on the standards and how they are implemented…’ (NMC 2023). There is a need for objective feedback on the views and experiences of nursing students that can provide a comparison between each field of nursing. This study attempts to provide such comparative evidence.

## 3 Methods

### 3.1 Aim and Research Question

The aim of the survey was to compare the experiences of third‐year students studying different fields of nursing in being assessed against the NMC (2018a) Future Nurse Standards of Proficiency to see if there were any differences between the fields in: reported levels of confidence to practise; the type of placements experienced; the frequency and nature of barriers to achieving proficiencies; experiences of assessment by Practice Assessors and any specific proficiencies/topics that students would like to see included in their training.

### 3.2 Study Design

A cross‐sectional research design in the form of an anonymous online survey of third‐year nursing students completing their training across UK universities. A mixed‐methods design used both descriptive quantitative data, as well as a qualitative analysis of students' free text responses.

### 3.3 Settings and Participants

An email was sent to all senior nursing academics across UK universities via Jiscmail groups and academic networks, containing a link to the online survey (on Microsoft Forms) with study invite/information, requesting that they post it as an announcement for third‐year students on the Learning Management System (LMS) for their course (e.g., Blackboard; Brightspace etc.). This was sent towards the end of the summer term when most students had completed all placements. The emailed information explained that the survey was anonymous and would not identify any student's university. Nursing academics were not required to respond to the authors to confirm whether they had posted the survey link for students. The only geographical identifier included in the survey asked students to indicate which of the 14 regions across the United Kingdom they were studying in. As each region contains multiple universities, this maintained anonymity.

### 3.4 Data Collection

Students received an email notification through their course LMS with the study invitation and information, where they were able to click on a link to complete the anonymous survey, which was open to collect responses between July and September 2023. The Microsoft form contained 10 questions, 8 of which were closed‐ended/multiple choice and 2 were open text to allow further explanation of students' responses (see Table 1).

**TABLE 1:** Online survey questions.

1. Please could you indicate which region of the country you are studying in? (choice of 14 regions)			
2. Which field of Nursing are you studying? (choice of adult; mental health; children's; learning disability)			
3. To what extent do you feel the NMC proficiencies in your practice assessments have enabled you to confidently practice in your field of nursing?			
Not at all	To a small extent	To some extent	To a large extent
4. During your training have you been allocated to any placement that was not aligned to your field of nursing? YES/NO			
5. If answered YES—What type of placements have you experienced that were different to your field? (choice of adult; mental health; children's; learning disability)			
6. Did you experience any problems or barriers in achieving any of the NMC proficiencies in practice placements? YES/NO			
7. If answered YES—Where there any specific proficiencies you've had difficulty in getting signed off and what factors hindered you in achieving them? (Open free text answer)			
8. Did you feel that your Practice Assessor was always a registered nurse with appropriate equivalent experience for your field of practice? YES/NO			
9. If answered NO—Which field of Nursing was your Practice Assessor qualified in that differed to your field of study? (choice of adult; mental health; children's; learning disability; same field of nursing but problems with experience e.g., insufficient experience in some proficiencies to sign them off)			
10. Are there any specific proficiencies or content that you would like to see included in the NMC curriculum, that are not currently? (Open free text answer)			

The form was originally piloted with a group of 12 third‐year students. A single scale to rate student confidence to practise in their field used the same four responses as the UK's National Student Survey (NSS, Office for Students 2024). This was chosen as students are familiar with this format (having recently completed the NSS) and single‐item scales have been shown to perform well in measuring concepts such as confidence, competence and autonomy (Martela and Ryan 2024).

### 3.5 Ethical Considerations

Ethical approval for the study was granted by the University Ethics Committee after implementing procedures to ensure the anonymity of students and universities was maintained in any free text responses (e.g., immediate deletion of any revealing information), and that students understood they would be unable to withdraw consent after submitting responses.

### 3.6 Data Analysis

Analysis of categorical data from survey questions with multiple choice responses was conducted using SPSS 25.0 software. Chi‐square tests analysed differences in responses between the nursing fields to detect significance at the level of < 0.05.

A thematic analysis of the open text responses was conducted using the reflexive approach of Braun and Clarke (2013). Three researchers, each senior nursing academics from different universities (one adult nurse qualified, and two dual qualified adult and mental health nurses) conducted analysis of the data separately following the first five stages of Braun and Clarke's (2013) six stage approach: familiarisation with the data; initial coding; generating initial themes; developing and reviewing themes; refining, defining and naming themes. Triangulation of each researcher's analysis was achieved by meeting to review, discuss and agree the final themes. A separate, senior academic (dual qualified in Adult and Mental Health Nursing), experienced in conducting qualitative research supervised and reviewed this process to ensure compliance with best practice.

## 4 Results

### 4.1 Response Rate by Field of Nursing and Region

Responses were received from students in 10 of the 14 UK regions for adult and mental health nursing; from 4 regions for Children's Nursing and 3 regions for Learning Disability Nursing (see Table 2). Due to the anonymity of the survey responses, no further data was recorded on student characteristics or university of study. The low response rate of 17 learning disability students was insufficient to draw conclusions from quantitative data analysis, but these responses are included for transparency (see Table 2).

**TABLE 2:** Number of student responses by region.

UK region (total)	Number of responses by field			
	Adult	Mental health	Children	Learning disability
South‐East England (102)	33	59	10	
West Midlands (95)	31	54	7	3
Yorkshire and the Humber (74)	29	27	8	10
Greater London (71)	26	29	12	4
Scotland (41)	17	24		
East of England (37)	17	20		
North‐West (32)	14	18		
East Midlands (32)	12	20		
Wales (24)	11	13		
North‐East (23)	13	10		
Total (531)	203	274	37	17

### 4.2 To What Extent Do You Feel the NMC Proficiencies in Your Practice Assessments Have Enabled You to Confidently Practice in Your Field of Nursing?

**FIGURE 1:**
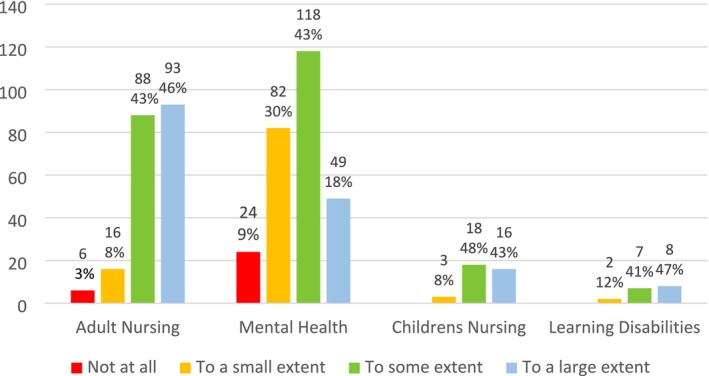
Responses to the question ‘To what extent do you feel the NMC proficiencies in your practice assessments have enabled you to confidently practice in your field of nursing?’.

The choices, ‘To a large extent’ and ‘To some extent’ are categorised as positive responses in the National Student Survey, whilst ‘To a small extent’ and ‘Not at all’ denote a negative response (Office for Students 2024). There were significant differences between the responses of mental health and adult nursing students (*p* = 0.001). Whilst 89% of adult nursing students positively reported that they felt confident to practise, only 61% of mental health nursing students rated this positively. Therefore, 39% of mental health students reported that the proficiencies had left them feeling unconfident to practise in their field vs. 11% of adult nursing students (See Figure 1).

### 4.3 During Your Training Have You Been Allocated to Any Placement That Was Not Aligned to Your Field of Nursing?

**FIGURE 2:**
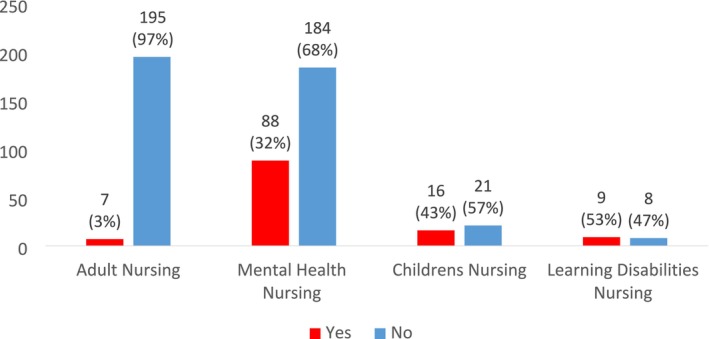
Responses to the question ‘During your training have you been allocated to any placement that was not aligned to your field of nursing?’.

Significantly greater proportions of mental health (32%), children's (43%) and learning disabilities (53%) students reported that they had been allocated to a placement outside of their field of practice compared to 3% of adult nursing students (*p* = 0.000; see Figure 2). The most commonly reported placement outside of the student's field of practice was adult nursing, comprising 80% of these external placements for mental health nursing students and 87% for children's nursing students (see Table 3).

**TABLE 3:** Responses to the question ‘What type of placements have you experienced that were different to your field of nursing?’.

	Adult nursing students	Mental health nursing students	Children's nursing students	Learning disabilities (LD) nursing students
	*N* = 7	*N* = 88	*N* = 16	*N* = 9
Number of students allocated to each type of placement outside their field of practice	Mental Health 1 Children 5 LD 1	Adult 70 (80%) LD 13 (15%) Children 5 (5%)	Adult 14 (87%) LD 2 (13%)	Mental Health 4 Adult 2 Children's 2

### 4.4 Did You Experience Any Problems or Barriers in Achieving Any of the NMC Proficiencies in Practice Placements?

**FIGURE 3:**
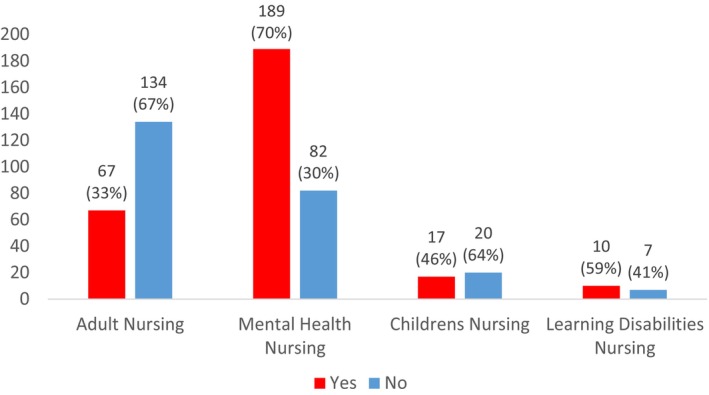
Responses to question ‘Did you experience any problems or barriers in achieving any of the NMC proficiencies in practice placements?’.

A significantly higher proportion of mental health nursing students (189; 70%; *p* = 0.000) reported barriers to achieving proficiencies compared to adult nursing students (67; 33%), children's nursing students (17; 46%) and learning disabilities nursing students (10; 59%) (see Figure 3).

### 4.5 Were There Any Specific Proficiencies That You Have Found Difficult to Get Signed Off in Practice?

The count of each proficiency mentioned in students' responses to this question revealed that those posing the most difficulties for students across all fields were invasive medical procedures. These were reported to a much greater extent by mental health nursing students, for whom managing blood transfusions, intravenous infusion pumps and catheters caused the most difficulties. Yet difficulties in the assessment of cannulation and venepuncture were also stated by both adult and children's nursing students, albeit to a lesser degree (see Table 4).

**TABLE 4:** Count of specific proficiencies that students found difficult to get signed off in practice.

Adult	Mental health	Children
*N* = 67^a^	*N* = 189^a^	*N* = 17^a^
Cannulation 13	Blood tranfusions 49	Cannulation 8
Venepuncture 10	IV infusion pumps 42	Venepuncture 7
Nasal/oral gastric tubes 7	Cannulation 38	Catheters 5
Blood transfusions 6	Catheters 32	Blood transfusions 3
Catheters 6	Nasal/oral gastric tubes 26	Cardiac assessment/ECG 1
Nasal/oral suctioning 4	Nasal/oral suctioning 13	Neurological assessment 1
Chest Auscultation 3	Venepuncture 12	Wound care/sutures 1
Medication management 2	Digital rectal evacuation 12	Digital rectal evaluation 1
Cardiac assessment/ECG 1	Cardiac assessment/ECG 2	Care for deceased 1
Neurological assessment 1	Neurological assessment 2	Challenging behaviour 1
Digital rectal evaluation 1	Wound care/sutures 2	

^a^ Students could report multiple proficiencies.

### 4.6 Did You Feel That Your Practice Assessor Was Always a Registered Nurse With Appropriate Equivalent Experience for Your Field of Practice?

**FIGURE 4:**
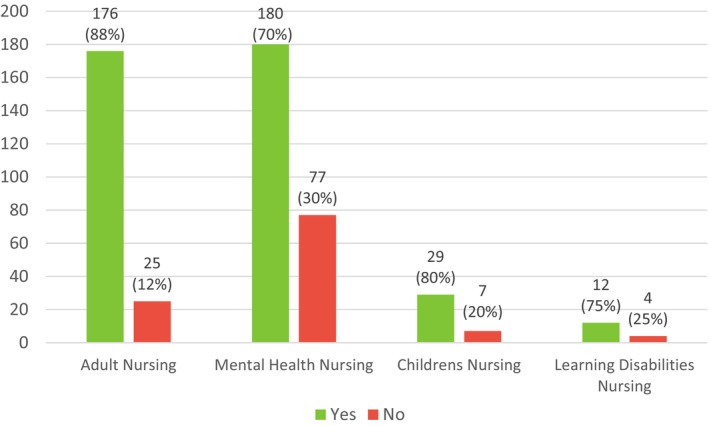
Responses to question ‘Did you feel that your Practice Assessor was always a registered nurse with appropriate equivalent experience for your field of practice?’.

A significantly higher proportion of mental health nursing students (77; 30%; *p* = 0.003) reported that their practice assessor did not have the appropriate level of experience compared to adult nursing students (25; 12%) and children's nursing students (7; 20%) (see Figure 4). Of those students who reported a problem with their assessor, the most common reason reported was that they were from the same field of nursing but lacked knowledge of certain proficiencies to sign them off (see Table 5). A significantly higher proportion of mental health nursing students (23; 29% *p* = 0.000) reported being allocated to a practice assessor from another field (adult nursing) compared to students from other fields for whom allocation to an assessor outside of their field was rare (see Table 5).

**TABLE 5:** Responses to the question ‘Which field of Nursing was your Practice Assessor (PA) qualified in that differed to your field of study?’.

	Adult nursing students	Mental health nursing students	Children's nursing students	Learning disabilities (ld) nursing students
	*N* = 27	*N* = 80	*N* = 8	*N* = 6
Same field but lacking knowledge of proficiencies to sign off	22	53	3	3
PA was an adult nurse		23	4	1
PA was a learning disabilities nurse	2	3	1	
PA was a mental health nurse	2			2
PA was a children's nurse	1	1		2

### 4.7 Are There Any Specific Proficiencies or Content That You Would Like to See Included in the NMC Curriculum, That Are Not Currently?

Fifty‐six percent of mental health nursing students responded, specifying a range of field‐specific mental health topics that they felt had not been included in their curriculum/practice assessments. The most requested of these (31%) were psychological therapies such as cognitive behavioural therapy (see Table 6). A lower proportion (36%) of adult nursing students requested specific competencies compared to the other fields, with 15 students requesting fewer practice hours/proficiencies rather than specific content topics (see Table 6). The most common topics requested by adult nursing students were communication skills or mental health‐related topics (see Table 6). A lower number of responses were received from children's nursing students, but these also requested child‐specific topics such as child development.

**TABLE 6:** Responses to the question ‘Are there any specific proficiencies or content that you would like to see included in the NMC curriculum, that are not currently?’.

Adult	Mental health	Children	Learning disability
*N* = 89^a^	*N* = 154^a^	*N* = 19^a^	*N* = 10^a^
Mental Health/communication **15** Reduced placement hours/renumeration/ability to change placements **10** Wound care **6** Learning disabilities **5** Anatomy/physiology **5** Fewer proficiencies **5** I.V. therapy **3** Cannulation **3** Neurological assessment **3** Equality & diversity **3** Simulation **3** Dementia **3** IT training **3** Research **3** Spiritual care **2** Arterial blood gases **2** Post‐op/emergency care **2** Resilience training **2** Team Handovers **2** Children related **2** G.P/primary care **2** Speak up support **2** District nursing **1** Palliative care **1** ECG **1** Full Respiratory Assess **1** Suction **1** Venepuncture **1** Health promotion **1** Sexual health **1** Relational safety **1** Other e.g., Provide guidance for assessors to sign off after discussion **3**	Psychological therapies (unspecified) **13** Cognitive behavioural therapy **11** Counselling **6** Dialectical behaviour therapy **5** Motivational interviewing **4** EMDR **3** Mindfulness **2** Psychodynamic **2** Solution focused therapy **1** Group therapy **1** MH medications **16** The law & MH **15** Risk assessment (prevention of suicide & self‐harm) **13** Therapeutic engagement/relationships **10** Psych therapy for psychosis **10** Psych therapy for personality Disorder/complex trauma **10** De‐escalation, conflict resolution & restrictive practices **9** MH diagnoses **8** Primary Care MH **6** MH team working **5** MH assessment **4** Psych therapy for bipolar disorder **4** Anxiety disorders **3** Eating disorders **3** ECT **3** Racism in MH care **2** Neurodiversity **2** Menopause & MH **2** Perinatal MH **2** Addictions **2** Neuropsychology **2** Others e.g., more MH simulation & training for assessors **5**	Child development/developmental psychology **4** Learning disability **2** Neurodiversity **2** Child anatomy **1** Blood gases on children **1** Observation equipment for children **1** PEGS feeding **1** NG tube training on children **1** Neonatal care **1** Eating disorders **1** Communication with children **1** Family dynamics **1** Psychological factors in nursing terminally ill children/child bereavement **1** Others e.g., No more proficiencies; staff wellbeing **3**	Mental capacity act **2** MH Act **2** Sensory stimulation **1** Safe use of restrictive practices **1** Neurodiversity **1** Psychological development **1** LD in all environments **1** Others e.g., No more proficiencies; request for medical placement **3**

^a^ Students could state multiple proficiencies/topics.

### 4.8 Thematic Analysis of Responses to Open Text Survey Question, ‘What Factors Hindered You in Achieving the Proficiencies?’

The number of respondents to the survey who completed this open text question totalled 388 out of 531 students (73%). The proportion of open text responses for each nursing field was: adult, 141 out of 203 (69%); mental health, 207 out of 274 (75%); children, 28 out of 37 (76%) and learning disability, 12 out of 17 (71%). From the thematic analysis of the responses, different themes emerged from each field.

#### 4.8.1 Themes of Mental Health Nursing Students

In contrast to adult nursing students, mental health students described the nature of the proficiencies themselves as a significant barrier to achieving them within their field of practice. The students expressed frustration that many of the proficiencies they were assessed against were not the vital proficiencies for their field, which produced three themes.

##### 4.8.1.1 Theme 1. Many Medical Proficiencies Are Not Practiced in Mental Health Settings

164 out of 202 mental health students expressed the view that many of the proficiencies are not very relevant to the field of mental health nursing and were not practised in their mental health placements. This was described as a major barrier to achieving the proficiencies, with many students having to ‘spoke out’ to medical placements to achieve them.

Some of the proficiencies were not relevant to mental health nursing and near impossible to get signed off as we would never be in a position or get the opportunity to do so.

As my field is mental health nursing we do not come across patients with catheters, infusion pumps, cannulation, enemas, manual evacuation. The proficiencies seem to be on a par with adult nursing which is a completely different field.

##### 4.8.1.2 Theme 2. Practice Assessors Vary in Willingness to Assess Medical Proficiencies

58 out of 202 mental health students described their practice assessors as lacking in knowledge/experience of certain medical procedures which they did not carry out in their mental health nursing roles and were therefore reluctant to assess these. Rather worryingly, several students stated that their assessor had been willing to sign these off without assessing them, based on the rationale that this would help the students because the proficiencies were not practised in mental health nursing.

These proficiencies… are very rarely carried out in mental health settings and the staff members in these settings are also not proficient in these areas, making it very difficult to get them signed off when we only have mental health placements.

PAs too anxious about comeback to sign off medical proficiencies they are not trained in.

I am very grateful to my last practice assessor in a mental health service who signed them all off without discussing it with me as they knew I wouldn't qualify otherwise. This shouldn't have to happen and it only seems to happen for mental health students because the proficiencies are not designed for us/our field.

##### 4.8.1.3 Theme 3. The Proficiencies Dilute Mental Health Nursing as an Identified Profession

14 out of 202 students expressed the view that the number of medical proficiencies meant that mental health proficiencies were missing from PARE and this diluted their profession of Mental Health Nursing.

There are many proficiencies related to adult nursing… although I appreciate that mental health nurses should be aware of these skills but making them as achievable proficiencies is unrealistic and takes away the focus of mental health nursing as an identified profession.

…the future nurse proficiencies are not fit for purpose due to not being field‐specific, I am training to be a specialised nurse with no specialised training.

…maybe the Epad needs to be specialised for each of the fields, so each nurse is actually unique and not a ‘general nurse’…

#### 4.8.2 Themes of Adult Nursing Students

The responses of the Adult Nursing students formed clear themes that highlighted contextual issues around the proficiencies, such as resources, relationships and policies, rather than factors concerning the proficiencies themselves.

##### 4.8.2.1 Theme 1. Extreme Differences in Relationships With Practice Assessors and Supervisors

21 out of 80 students who responded described disagreements/differences of opinion with supervisors/assessors and being on the receiving end of their expressions of stress and frustration. Some described a lack of willingness from their supervisors/practice assessors to engage with them, two students described bullying and two described racism.

There was an atmosphere of bullying on the ward in my last placement and my PA was an unpleasant person. When I asked for guidance she said I was a third‐year student and should show more initiative, but she told me to do things without explaining them properly…

My practice supervisor stated she hated teaching students… I did not get spoken to on the ward and was not able to take a power hour for study whilst there.

Whereas 10 students completed this free text field to explain that they had experienced good support from their supervisor/assessor. These students described their practice assessors as being supportive and helpful, which enhanced their learning experience.

Most placement supervisors and assessors were very supportive.

I've always got them signed off and had good support from my last practice assessor who admitted they weren't an expert on all of them but took the time to discuss lots with me…

##### 4.8.2.2 Theme 2. Insufficient Resources and Opportunities

20 out of 80 students who responded described insufficient time due to a lack of staffing as a barrier. Practice assessors did not have the time to assess/sign off their proficiencies due to the lack of these resources. Students also described not being on the same shift as their assessor or not being allocated to another assessor when their assessor was off sick or on annual leave. Several students described being used as an extra member of staff rather than as a learner.

I was used like a HCA on wards and my supervisor did not have the time to arrange learning experiences for me so I did not get to carry out or experience all proficiencies.

My Practice Assessor went on Annual Leave and then was off sick so I had to get someone else to sign off my proficiencies at final interview after my placement had actually ended and they didn't know me.

Student nurses should get paid some money if they are going to be counted as part of the numbers due to the shortage of nursing staff!

##### 4.8.2.3 Theme 3. Prohibitive Policies and Lack of Permission

15 out of 80 students described NHS trust policies that prevented them from carrying out certain invasive procedures as students. This was expressed as a source of frustration as some practice assessors were also unwilling to sign off these proficiencies even after a detailed discussion of the student's knowledge and understanding, as they had not observed the student perform the procedure.

The trust does not allow students to do cannulation—a basic practice once we are qualified.

Local policies within the trust made it difficult to achieve some proficiencies—for example blood transfusion and insertion of ng tube…

Some assessors prefer not to sign you off if they haven't seen you directly complete the proficiency. This can be really frustrating as it feels as though they don't believe you…

#### 4.8.3 Themes of Children's Nursing Students

##### 4.8.3.1 Theme 1. Children's Nurses Are Prohibited From Demonstrating Some Proficiencies

Although fewer children's nursing students completed this free text question with only 28 responses, a clearly identified theme across 12 of these was the assertion that qualified children's nurses do not perform certain invasive medical procedures in clinical practise, as these are carried out by doctors. This meant that some practice assessors would not assess these proficiencies for students.

As a child nurse not all proficiencies are applicable, for example catheterisation, in the trust umbrella we are under this is done by the doctors and not even advanced nurses.

ECG's and end of life care are not commonly done/seen in children's nursing practice. venepuncture and cannulation and catheterisation—trust policies don't allow students to complete these proficiencies.

## 5 Discussion

Mental health nursing students reported significantly lower levels of confidence to practise in their field due to the generic proficiencies compared to the high confidence reported by adult (*p* = 0.001), and to some extent children's nursing students (see Figure 1). There was a significant difference between fields in the type of placements experienced (*p* = 0.000), with adult nursing students remaining within their field, but mental health and children's nursing students requiring adult nursing placements (see Table 3) because proficiencies in invasive medical procedures are not routinely practised in their field. As a result, significantly more mental health nursing students reported barriers to achieving the proficiencies in practice (*p* = 0.000), and that their practice assessor did not have the required knowledge/experience of medical procedures compared to the other fields (see Figure 4). Twenty‐three mental health nursing students reported that their practice assessor was an adult nurse, supporting the claim that mental health nursing students have to chase medical procedures requiring placements in adult nursing settings to be signed off (Haslam 2025).

There were clear differences between the fields in the topics students had not received teaching on and would like to see included. Both mental health and children's nursing students requested field‐specific content/proficiencies, with psychological therapies most requested by mental health students, and factors related to child development highlighted by children's nursing students (see Table 6). Interestingly, adult nursing students most requested proficiencies were also communication skills to address patients' mental health, indicating that these skills are excluded from generic curriculums and practice assessments.

### 5.1 Limitations

The responses from 274 mental health and 203 adult nursing students' responses were sufficient to address the research questions, but 37 responses from children's nursing students only provide some indications for this field, and 17 responses from learning disabilities nursing students are insufficient to draw conclusions. It is not possible to know how many students were sent the survey invite overall, so the response rate is not available; however, 531 responses is obviously a small proportion of the total number of third‐year nursing students across the United Kingdom. This raises the problem of non‐response bias in student surveys, in which only students with more extreme experiences/opinions may respond (Standish and Umbach 2019). Utilising nursing academic networks and university departments was a useful way of accessing students, but the authors were made aware of gatekeeping practises at some universities which prevented the survey from being shared with students and may have introduced a different kind of non‐response bias. Some survey questions were focused on problems/barriers rather than positive aspects of the proficiencies, which is an explicit bias. Yet the aim of the study was a comparison between the experiences of mental health nursing students and those of the other fields in relation to barriers that had been reported by academics (e.g., Bifarin et al. 2024; Haslam 2025) rather than a general assessment of how the NMC proficiencies are experienced. The qualitative thematic analysis of limited open text survey responses lacks depth and is not an ideal application of this method (Braun et al. 2021), but specific descriptive qualitative data was required to explain the categorical responses.

### 5.2 Implications for the Mental Health Nursing Field

The feedback from mental health nursing students indicates that the shift to generic proficiencies is having unintended negative effects. Students are concerned that the skills required for mental health nursing (e.g., psychological/talking therapies) are underrepresented in their practice assessment documents. They feel reduced to referral agents rather than skilled practitioners, which damages their confidence, sense of professional identity and worth, as expressed in one student's comments:

…we are supposed to have skills in therapies which are in Annexe A, but they are not on the e‐pad other than ‘actively participates in the safe referral of people to other professionals such as talking therapies’ so it's actually saying that we don't have these skills, we just refer patients to those who do—it's insulting.

The generic standards aim to unify and raise the level of care across all fields, but in practise, mental health nursing students report that they prioritise medical skills over mental health expertise. This feedback appears to support the arguments of academics that the loss of specialist mental health nursing skills from curriculums devalues the mental health nursing profession and threatens the quality of future mental health care (Connell et al. 2022; Haslam 2023; McKeown 2023; Warrender et al. 2023).

This initial feedback from UK students appears to mirror the Australian experience where students on generic nurse training courses have also reported low levels of confidence in interacting with people suffering from mental health problems (McGough and Heslop 2021). Descriptions of the mental health nursing profession being diluted also echo those of Australian academics (Hercelinskyj et al. 2014; Happell 2014). The longer‐term impact of generic nurse training in Australia has seen nurses without mental health credentials working in mental health services (Sheehan et al. 2025), and an under‐utilisation of therapeutic communication skills in practise (Hurley et al. 2022). The effect has been significant workforce shortages due to nurses' reluctance to work in such a devalued field (Lakeman et al. 2024; Hurley and Lakeman 2021; Lakeman and Molloy 2018). These lessons seem to have been ignored, and it is concerning that qualifying mental health nursing students in the United Kingdom are now indicating similar experiences. Comparable challenges are being experienced in other countries such as Belgium where the mental health speciality has been discontinued in the nurse training programme (Desmet et al. 2025). Steps towards generic nurse training have already been implemented in the United Kingdom—since 2019 the NMC has allowed generically‐trained international nurses to register as Mental Health Nurses regardless of prior experience after passing a test of competence (NMC 2024).

The generic ‘Future Nurse’ standards of proficiency (NMC 2018a) are set out in two sections for practice assessment: Annexe A includes communication and relationship management skills, which are interpreted as basic values across the programme (Evans 2024) rather than listed as explicit proficiencies in practice assessment documentation. Annexe B contains a list of medical procedures that must be signed off by practice assessors and are therefore the focus of practice assessments. Mental health and adult nursing students both identified proficiencies from Annexe A as missing from their training, stating they would like to see them included in the curriculum. The clear implication of such feedback is that a revision of curriculum guidance and practice assessment documentation is needed to ensure that the communication and relationship management proficiencies of Annexe A are made more tangible, explicit and mandatory.

The UK Government and NHS England set out a commitment to achieve ‘Parity of Esteem’ in healthcare—the principle by which mental health must be given equal priority to physical health (UK Parliament 2020). This principle was enshrined in law by the Health and Social Care Act (2012). Whilst mental health nurses need to care for the physical health problems of people with mental illness (Nash 2022), students report that the principle of parity is not being implemented in nurse training, as the proficiencies prioritise physical care over mental health.

### 5.3 Implications for Practice Assessors

The survey feedback indicates that new field‐specific guidance may also be needed for practice assessors, as student feedback indicated extreme differences in supervisors/assessors' attitudes, supporting existing evidence that assessors in different fields have different priorities when assessing students (Painter and Bond 2023). Mental health nursing students reported that assessors varied in their approach from a refusal to sign off any medical proficiencies not routinely practised in mental health settings to (worryingly) passing them without assessment because they do not view medical proficiencies such as venepuncture and cannulation as part of mental health practise. Adult nursing students reported relational problems with their assessors as the most common barrier to achievement rather than the content of the proficiencies themselves. Existing evidence suggests that training for practice assessors on implementing Standards for Student Supervision and Assessment (NMC 2018c) and using/interpreting practice assessment documentation has been inconsistent (Addis and Loughrey 2025), and students' reported experiences suggest that further research is needed comparing the experiences of practice assessors across the fields of nursing to help inform further guidance on field‐specific practice assessments.

### 5.4 Implications Across the Other Fields of Nursing

The generic NMC proficiencies appear to be providing adult nursing students with a high level of confidence to practise successfully, matching the positive views of adult nursing educators (Whaley et al. 2024) and enabling them to remain within their field of practice throughout training. However, the limited feedback from children's nursing students indicates they may experience barriers to achieving proficiencies in medical procedures not performed on children's wards and requiring placements in adult nursing settings. This supports existing evidence that UK universities are applying the standards in a generic way which focuses on adult nursing proficiencies (Royal College of Nursing 2024). Children's nursing students also requested field‐specific topics not covered sufficiently in their training such as child development, which fits with published concerns that child‐specific knowledge and proficiencies are being lost from training courses (Glasper and Charles‐Edwards 2002). The low number of responses from learning disabilities nursing students in comparison to the other fields supports the Royal College of Nursing (2024) analysis that many parts of the United Kingdom have become ‘learning disability nurse deserts’, with collapsing student numbers, courses closing and a 43% drop in the number of learning disability nurses employed by NHS Trusts since 2010.

## 6 Conclusions

Striking differences were reported between nursing fields in students' experiences of assessment against the generic proficiencies. Mental health nursing students reported that the shift to generic proficiencies is having unintended negative effects, as practice assessment documentation and curriculums prioritise physical health/medical skills over mental health‐specific expertise, causing extreme differences in practice assessors' attitudes and behaviours. The findings suggest an urgent review and rebalancing of the proficiencies is needed to ensure UK universities make proficiencies in therapeutic communication and relationship management explicit in practice assessment documentation and curriculums as standard. Further research is also required to compare the experiences of practice assessors across the fields of nursing.

## 7 Relevance for Clinical Practice

Lower confidence to practise and exclusion of mental health content and proficiencies from curriculums and practice assessments may indicate that generic application of the NMC standards of proficiency in the United Kingdom is producing mental health nurses with fewer proficiencies in therapeutic communication and reducing the quality of care provided in mental health services. There is a need for mental health nurses in the United Kingdom to identify and articulate the specific proficiencies of their field and ensure these elements of Annexe A are made explicit and mandatory in practice assessment documentation. Further guidance may be required for practice assessors on how field‐specific practice assessments can be contextualised and proficiencies signed off to ensure that students have, “the ability to undertake these procedures at an appropriate level for their intended field(s) of practice” (NMC 2018a).

## Ethics Statement

Ethical approval for the study was granted by the University of Staffordshire's Ethics Committee.

## Conflicts of Interest

The authors declare no conflicts of interest.

## Data Availability

The data that support the findings of this study are available on request from the corresponding author. The data are not publicly available due to privacy or ethical restrictions.
